# Laparoscopy-assisted peritoneal dialysis catheter placement using a modified minimally invasive approach: A retrospective observational study

**DOI:** 10.1097/MD.0000000000035814

**Published:** 2023-10-27

**Authors:** Necmi Bayraktar, Fazil Tuncay Aki

**Affiliations:** a Burhan Nalbantoğlu State Hospital, Nicosia, Northern Cyprus, Turkey; b Hacettepe University School of Medicine, Urology Department, Ankara, Turkey.

**Keywords:** minimal invasive dialysis catheter placement, modified laparoscopic approach, peritoneal dialysis, peritoneal dialysis catheter placement, standard laparoscopic peritoneal dialysis catheter insertion

## Abstract

Peritoneal dialysis is a reliable and effective treatment for end-stage kidney disease. However, inadequate catheter insertion can lead to mechanical dysfunction, which remains an unresolved problem. In this study, we present the initial results of a modified laparoscopic approach. This study included 38 patients who underwent peritoneal dialysis using a modified laparoscopic approach. During the procedure, a single laparoscopic trocar was employed, and peritoneal entry was performed using a percutaneous pull-apart sheath/dilator. To minimize the risk of complications, the free catheter portion was kept short in the peritoneum. The modified method was guided by proven recommendations of the standard laparoscopic technique. The mean operation time was recorded as 24.28 ± 15.5. The mean hospitalization was found to be 1.20 ± 0.72 days. The postoperative morbidity was 26.3%. The mechanical dysfunction rate was 5.26%. The median follow-up time was 20.4 ± 17.14 months. The median peritoneal dialysis catheter-free survival was 25.96 ± 4.02 months. The catheter-free survival rate was 92.11%. The modified laparoscopic approach has been demonstrated to be a safe and effective option, and initial studies have indicated that it offers several benefits over traditional methods, including a straightforward procedure with a brief duration, minimal complications, and brief hospital stay.

## 1. Introduction

Peritoneal dialysis is an effective and inexpensive treatment compared with hemodialysis for end-stage renal failure.^[1]^ Approximately 11% of dialysis patients receive peritoneal dialysis.^[2,3]^ Northern Cyprus has a population of approximately 400,000. These are the available options for treating end-stage renal disease, including hemodialysis, peritoneal dialysis, and kidney transplantation. Each year, approximately 400 patients receive dialysis treatment at 4 state-operated centers. Patients requiring dialysis may opt for peritoneal dialysis, which involves inserting a catheter into the peritoneal cavity to facilitate efficient fluid exchange and improve outcomes.^[4,5]^ A peritoneal dialysis catheter was placed during the open surgery. Currently, laparoscopic methods are widely preferred.^[3]^

Although percutaneous, peritoneoscopic, and laparoscopic techniques have lower complication rates than open surgery, significant complication rates have been reported. Various studies have reported differing complication rates, which can result in treatment termination due to complications associated with peritoneal dialysis catheter placement. For successful and effective catheter function in laparoscopic advanced peritoneal dialysis catheter placement, evidence of the success of advanced laparoscopic methods such as omentopexy and omentectomy, as well as adhesion lysis, rectus sheath tunneling, and preservation of peritoneal integrity, has been clearly demonstrated.^[6]^ In our experience, not every patient requires placement of a peritoneal dialysis catheter using an advanced laparoscopic method. Our peritoneal dialysis treatment outcomes were negatively impacted by the high complication rates associated with peritoneal dialysis catheters placed using the standard laparoscopic technique. Consequently, the number of patients undergoing peritoneal dialysis has decreased. Recent studies and evidence on low-site peritoneal dialysis catheter placement have encouraged us to modify our approach.^[7]^

We aimed to enhance the success rate by implementing straightforward modifications that capitalize on the advantages of the standard laparoscopic approach, ultimately reducing the mechanical complications.

## 2. Material and methods

### 2.1. Study design and data collections

Between 2018 and 2022, 38 patients who underwent peritoneal dialysis catheterization using the modified laparoscopic method were retrospectively evaluated. The evaluation included a comprehensive assessment of clinical and operative characteristics as well as an analysis of postoperative infectious and mechanical complications and follow-up results. The findings of this evaluation provide valuable insights into the efficacy of the modified laparoscopic method for peritoneal dialysis catheterization and its associated outcomes.

### 2.2. Inclusion and exclusion criteria

Before the operation, the patients were informed of the procedure, and informed consent was obtained. Exclusion criteria included a history of previous abdominal surgery, pediatric patients, and those who underwent revision surgery for mechanical dysfunction. Life expectancy was not an exclusion criterion for this study.

### 2.3. Data analysis

Continuous variables were expressed as mean ± standard deviation (SD). The recorded data are presented as descriptive statistics and percentages (N and %, respectively), including patient demographics, operation duration, postoperative events, hospitalization, and early and late complications. Catheter survival and outcomes were evaluated using Kaplan–Meier survival analysis. The obtained data were analyzed using the statistical package program SPSS.inc Windows version 24.0.

### 2.4. Ethical considerations

This study was conducted in accordance with the guidelines of the Declaration of Helsinki and ethical approval was obtained from the Ethics Committee of the Northern Cyprus Ministry of Health (Number: YTK.1.01-EK25/22).

### 2.5. Peritoneal dialysis catheter placement protocol

#### 2.5.1. Modified laparoscopic technique for peritoneal dialysis catheter insertion (Fig. 1).

**Figure 1.**
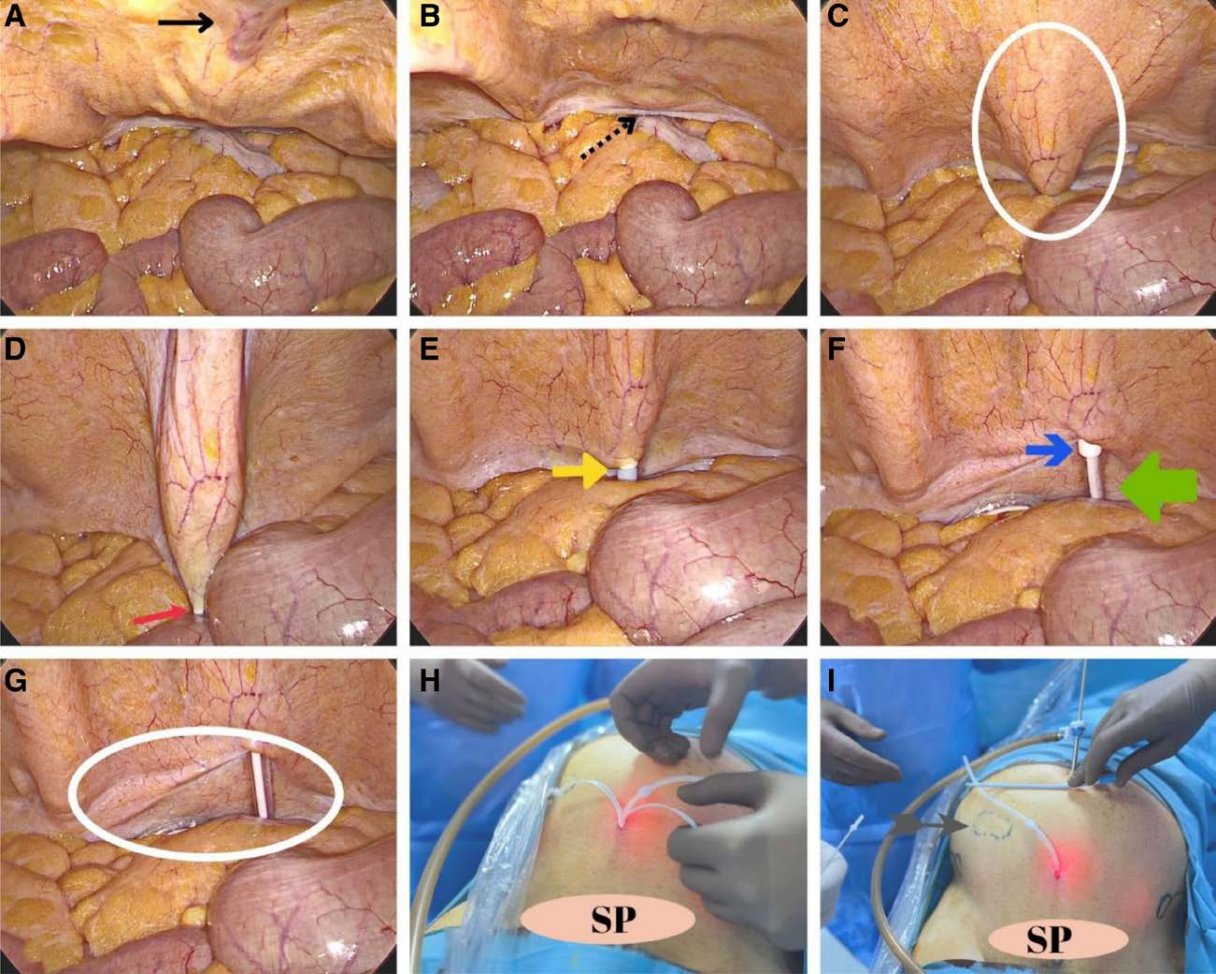
Modified laparoscopic approach. (A) The location of the skin incision and the starting point of the rectus sheath tunnel are indicated by black arrows. (B) A black arrow with dashed lines indicates the target entry point into the peritoneum. (C) In the white circle, the pull/apart sheath and dilator were observed laparoscopically. (D) The image shows the insertion of the pull/apart dilator into the peritoneum at a 70–90-degree angle, with the tip of the trocar indicated by the red arrow. (E) Under laparoscopic observation, the pull/apart dilator was advanced into the Douglas cavity until the white sheath was visible, as indicated by the yellow arrow. (F) The green arrow shows the advancement of the peritoneal dialysis catheter into the Douglas space, whereas the blue arrow shows the sheath of the pull/apart dilator, extending into the peritoneum. (G) The white circle is a peritoneal dialysis catheter with a short segment remaining inside the abdomen. (H) Removal of the pull/apart dilator and positioning of the peritoneal dialysis catheter. (I) The image shows the spot on the skin where the peritoneal dialysis catheter exits and the appendectomy scar is located, which is marked by a gray arrow. SP: symphysis pubis.

While the patient was in the supine position, a pneumoperitoneum of 12 to 14 mm Hg was created using a Verres needle under general anesthesia. A 5 mm optical trocar was inserted at the umbilicus level, and the abdominal cavity was examined using a 30-degree laparoscope. The patient was then positioned at a 30-degree angle and underwent diagnostic laparoscopy (Fig. 1A and B), which was accessed by performing sharp and blunt dissections in the rectus fascia through a 1 cm incision made 5 to 6 cm from the pubic symphysis.

The rectus fascia was accessed through a midline incision and the pull-apart sheath/dilator was placed 1 cm lateral to the midline. The sheath/dilator was used to pass the fascia from the preperitoneal space between the bladder into the Douglas space (Fig. 1C), done under direct vision.

The pull-apart sheath/dilator was positioned vertically and the rectus fascia was punctured with a trocar tip. The sheath/dilator was advanced—3 to 4 cm through the rectus sheath and monitored laparoscopically (Fig. 1D). At the puncture site, the pull-apart sheath/dilator was placed upright to stretch and enter the peritoneum, while protecting the tunnel (Fig. 1E). The dilator was then removed and the peritoneal dialysis (PD) catheter was inserted through the sheath (Fig. 1F–H). After detaching the trocar sheath, the PD catheter was rotated, guided into the abdomen, and inserted into Douglas space.

A distal cuff was placed at the entrance of the rectus fascia and the skin exit site was incised to a depth of 3 to 4 mm. The skin exit site was located on a line drawn parallel to the right or left side of the abdomen, 2 to 3 cm below the navel. The patient was then placed 3 cm below the navel. The subcutaneous route was performed using a blunt technique by advancing over the fascia with the metal trocar guide of the PD catheter (Fig. 1I).

No permanent or dissolving sutures were used during surgery. If there were no dense intra-abdominal adhesions, they were released using a 5 mm energy device. The pull-apart sheath allows the use of a 5 mm laparoscopic instrument. However, if adhesion is severe, an additional trocar may be placed to release it. The peritoneal dialysis technician confirmed the fluid inlet and outlet twice, while maintaining sterile conditions. The first fluid flow test was performed under increased intra-abdominal pressure (12 mm Hg), and the second follow-up was performed after correcting the Trendelenburg position and evacuating intra-abdominal carbon dioxide gas.

The modified minimally invasive laparoscopic approach involves laparoscopy to assist the placement of a percutaneous dialysis catheter.

#### 2.5.2. In the standard laparoscopic technique.

In the supine position, under general anesthesia, pneumoperitoneum (12–14 mm Hg) is induced using the Verres or open technique and surgery is initiated. Preferably, a 5 mm optical trocar was placed at the level of the umbilicus and the abdominal cavity was inspected using a 5 mm 0-degree laparoscope. Subsequently, the patient was placed in the Trendelenburg position and diagnostic laparoscopy was performed. Subsequently, an extra 5 mm trocar of 5 mm is inserted under direct vision from the exit site of the PD catheter. The 5 mm extra port inserts an additional 2 to 3 cm below the umbilicus to the right or left of the umbilicus. This trocar was advanced 2 to 4 cm into the preperitoneal area between the anterior and posterior sheaths of the rectus muscle and passed into the peritoneum from the midline. Subsequently, the PD catheter was inserted through the paraumbilical trocar into Douglas cavity. If adhesions were detected in the abdomen, a second working port was placed, and the adhesions were released using a monopolar electrocautery or energy device. An additional trocar was not required in the absence of intra-abdominal adhesion. However, if there is a problem with catheter position, an additional trocar should be placed to bring the PD catheter to the appropriate position. After ensuring that the catheter was appropriately placed, the paraumbilical trocar was removed. Subsequently, a subcutaneous tunnel was created using a catheter needle and the proximal cuff of the catheter was placed in the tunnel. The distal cuff should remain between the 2 rectus sheaths. The abdomen was emptied, the catheter was placed under direct vision, and its position determined. The rectus sheath was then closed using absorbable sutures. Simultaneously, the skin was closed with resorbable monofilament sutures.

A summary of the main points and differences of the modified approach is provided below.

- The laparoscopic procedure is completed using a single trocar.
- Peritoneal entry is performed with a percutaneous pull-apart sheath/dilator.
- Rectus sheath tunneling is considered an indispensable principle in each case.
- The catheter is positioned in the peritoneum to fit completely into the Douglas cavity.
- Care was taken to keep the free catheter part short in the peritoneum, thus limiting its floating.
- No suture fixation is performed within the skin or peritoneum.

## 3. Results

From 2018 to 2022, a modified laparoscopic peritoneal dialysis catheter was inserted in 38 patients (29 [76.3%] men and 9 [23.7%] women) eligible for peritoneal dialysis. The primary kidney disease distribution of the patients was as follows: unknown, 9 (23.7%); hereditary kidney disease, 7 (18.4%); diabetic nephropathy, 6 (15.8%); glomerulonephritis, 6 (15.8%); hypertensive kidney disease, 5 (13.2%); systemic disease, 4 (10.5%); and post-traumatic kidney disease, 1 (2.6%). The average age of the patients was 58.4 ± 13.18 years, and the average body mass index was 27.88 ± 4.84. Sixty percent of the patients had hypertension, 15% had diabetes, and 10.5% had heart failure. Fourteen patients (35.1%) had a history of abdominal surgery. Abdominal access was achieved using a single trocar (optic) in all patients except for 4 patients with severe abdominal adhesions.

The preoperative demographic characteristics, perioperative information, and postoperative records of the patients are shown in Table 1. The mean operation time was calculated as 24.28 ± 15.5 minutes. The operative time did not include the patient’s preparation or the start of anesthesia. No mortality was observed for any patient. A 10 mm trocar was used as an optical trocar in 21 patients (55%). Lysis of the adhesion was performed in 6 patients, and hernia repair was performed in 2 patients. Catheter fixation, omental fixation, and omentectomy were not performed in any patient. Before the end of the surgery, the inlet and outlet fluid flows were checked for 250 mL. Peritoneal dialysis was initiated in all patients within 4 days. No exit-site leakage was observed in any patient during the first 30 days. The mean hospitalization time was 1.20 ± 0.72 days postoperatively.

**Table 1:** Descriptive statistics and outcomes of the modified laparoscopic PD catheter insertion.

Characteristics	Total number of patients (N)	Results
Total patients	38	
Age (years, mean ± SD)		58.4 ± 13.18
*Gender*		
Male	29	76.3%
Female	9	23.7%
BMI (kg/m2, mean ± SD)		27.88 ± 4.84
Operation time (minutes, mean ± SD)		24.28 ± 15.5
Hospitalization (days, mean ± SD)		1.2 ± 0.72
*Preoperative risk factors*		
Hypertension	23	60.5%
Diabetes	6	15%
Heart failure (EF < 40%)	4	10.5%
Previous abdominal surgery	17	44.7%
*Additional procedure performed*		
Adhesiolysis	6	15.7%
Hernia repair	2	5.26%
Omentectomy	Nil	
Omentopexy	Nil	
Postoperative morbidity	10	26.3%
Cardiac event	1	2.6%
Trocar hernia	6	15%
Exit site leakage (ESL) within 30 days	Nil	
Peritonitis within 2 weeks	1	2.6%
Mortality	Nil	
Technical complications	15	39.4%
Bowel perforation	Nil	
Hemorrhage	2	5%
Ultrafiltration failure	2	5%
Omental wrapping	2	5%
Catheter migration	Nil	
Early leakage within 2 weeks	Nil	
Late leakage	2	5%
Transient obstruction	3	7.8%
Catheter reinsertion	2	5%
Tunnel infection	Nil	

BMI = body mass index, EF = ejection fraction, ESL = exit site leakage, SD = standard deviation;

Morbidity was documented in ten patients. Bleeding did not require transfusion in 2 patients (Clavien-Dindo grade 1), cardiac events in 1 patient (Clavien-Dindo grade 4), hernia in the optic trocar region in 6 patients (Clavien-Dindo grade 3b), and peritonitis (Clavien-Dindo grade 1) in 1 patient^[8]^ within 2 weeks. Hernia occurred at the trocar site in 6 patients who used a 10 mm optical trocar. However, none of the patients underwent hemodialysis after hernia repair. No hernia occurred in any patient using a 5 mm optical trocar was used. Intestinal perforation was not observed in the patients in the early and late postoperative periods. Postoperative bleeding occurred in 2 patients. No surgical intervention or blood transfusion was required. These patients were followed-up and treated with sequential washing and early peritoneal dialysis. Both the patients with bleeding had a history of antiplatelet drug use.

During follow-up, ultrafiltration failure was detected in 2 patients. Therefore, the patient was switched to hemodialysis. Leakage occurred in the late period in 2 patients. The patients were followed up medically, and peritoneal dialysis was continued. Transient obstruction occurred in 3 patients. However, the obstruction was resolved using interventional methods without surgical intervention. No additional surgical intervention was required. Two patients underwent surgical intervention for obstruction. In these patients, mental wrapping was determined to be the cause of obstruction. The peritoneal dialysis catheters were removed and reinserted.

The median follow-up period was 20.38 (SD ± 17.14). Seven patients died during the follow-up period. One patient who developed peritonitis after surgery switched to hemodialysis. Of the 7 patients who switched to hemodialysis, only 2 switched because of mechanical dysfunction. Three patients switched to hemodialysis for psychological reasons, and 2 were due to ultrafiltration failure.

**Figure 2.**
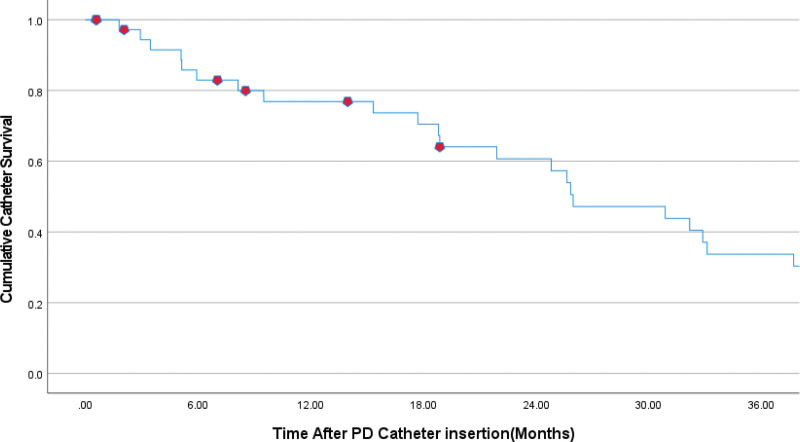
PD mechanical survival rate during the follow-up period.

The median PD catheter mechanical survival time was 25.96 ± 4.02 (95%CI) months. The PD mechanical survival rate was 92.11%, and The Kaplan–Meier curve is shown in Figure 2.

## 4. Discussion

Peritoneal dialysis catheters placed through a modified laparoscopic approach have increased the proportion of patients receiving peritoneal dialysis catheter treatment in Northern Cyprus. The low incidence of early and late complications is a decisive factor in the choice of peritoneal dialysis.

PD catheter placement using the open and percutaneous techniques was performed with blind vision. However, while laparoscopy enables observation of the inside of the abdomen, it also makes it possible to intervene in adverse events that may affect the PD catheter survival. Catheter survival is compromised by mechanical issues, such as migration, exit site leakage, tunnel infection, and omental wrapping.^[9]^ Although, laparoscopic method is the most common method for insertion of a peritoneal dialysis catheter worldwide.^[10]^ Unfortunately, there is no worldwide standardization of standard or advanced laparoscopic PD catheter insertion.^[11]^ Catheter survival can be negatively affected by both early and late complications, arising from surgical trauma and improper positioning.^[9]^

Our enhanced approach combined the benefits of both laparoscopic and percutaneous procedures. It strives for direct visualization through laparoscopy, with minimal tissue damage using the percutaneous method. Conventional laparoscopic techniques often report exit-site leaks and tunnel infections; however, percutaneous approaches are uncommon.^[12]^ Nevertheless, mechanical dysfunction and organ injury due to blind access are more prevalent in percutaneous techniques. The modified method reduces complications and mechanical dysfunction based on the outcomes of the maneuvers and approaches described. The need for anesthesia and advanced laparoscopic skills are the disadvantages associated with the insertion of a peritoneal dialysis catheter. Between the period of insertion of the standard laparoscopic PD catheter and the period of insertion using the modified method, the number of patients undergoing peritoneal dialysis in Northern Cyprus doubled.

Laparoscopic lysis of the adhesions is recommended to reduce catheter dysfunction.^[7]^ In adhesions that do not minimize peritoneal capacity and disrupt its integrity, adhesions can be released by passing a 5 mm ultrasonic shears through the pull-apart sheath/dilator without the need for an additional trocar, which will prevent the insertion of a suitable PD catheter in the Douglas pouch in the pelvic region.^[8]^ The larger the number of compartments and the greater the area occupied, the greater is the surgical intervention required. Additional surgical interventions are associated with higher risks of bleeding, fibrin formation, and catheter occlusion. However, in a study by Keshvari et al, there was no difference in catheter function in patients with a virgin abdomen who had undergone previous surgery.^[13]^ Therefore, the correct insertion of the catheter into the Douglas pouch takes precedence over excessive lysis.

The use of sutures to secure the catheter in place prevents it from moving and causing malfunction, thus reducing the risk of catheter dysfunction.^[14]^ However, catheter removal is difficult, and there is an increased risk of severe adhesions and internal hernia.^[15]^ Frost et al recommended that “proper rectus sheath tunneling is key to reducing catheter migration,” not suture fixation.^[15]^ If the PD catheter is positioned as a long piece in the abdomen, it will gain floating motion when the abdomen is filled with dialysate. In our approach, the catheter section in the abdomen was kept short and the mobility of the catheter was restricted. This eliminated the need for catheter fixation using sutures. No additional trocar entry was required for catheter fixation or positioning.

Rectus sheath tunneling is considered superior to catheter fixation, because it eliminates the time required for suturing to prevent catheter migration.^[16]^ Our approach aimed to shorten the intraperitoneal segment of the pelvis by creating a pre-peritoneal tunnel and an appropriate entry angle into the peritoneal cavity. This prevents catheter migration. A minimal preperitoneal dead space was created when a pull sheath/dilator was used as a peritoneal access incubator. The diameters of the pull-apart sheath/dilator were 5.3 mm, that of the peritoneal dialysis catheter was 4, and 7 mm, respectively. In contrast, the outer diameter of the blunt trocar used in the traditional methods was 8 mm. The pull-apart sheath/dilator passes bluntly through the fascia and extends into the pelvis, following minimal preperitoneal damage. Owing to its diameter and structural features, it does not disrupt the integrity of the peritoneum and leaves almost no space for leakage. In addition, the pull sheath/dilator was designed to place a peritoneal dialysis catheter, and trocars and other instruments used in traditional methods have been adapted for this procedure.

Omental wrapping is known to cause catheter-related dysfunctions. Even in open surgeries, omentectomy was performed through the incision and the upper abdominal wall was fixed with sutures. Both omentectomy and omentopexy can be laparoscopically performed. However, the risk of bleeding requires a long operative time and advanced laparoscopic experience and skill. Ogunc et al routinely used a laparoscopic omental fixation (omentopexy) technique.^[9]^ Ogunc et al reported that the method was effective and successful compared to the open surgical procedure and that catheter survival was better due to omental wrapping. The omentopexy technique has been shown to avoid omental wrapping; however, it requires the insertion of multiple working trocars and relies on the skill of the surgeon performing the laparoscopic surgery. This causes more abdominal wall trauma, increased bleeding risk, adhesions, and prolonged operative time. In addition, hospitalization time may increase, which is not cost-effective.^[6,11,17–19]^ According to our results, this modified laparoscopic PD catheter insertion achieved a technical dysfunction rate of 5% omental wrapping when the PD catheter was correctly inserted into the Douglas pouch.

Baksi et al investigated the use of omentectomy to reduce catheter malfunctions. They found that the relationship between laparoscopic omentectomy and catheter dysfunction was weak, especially in patients who underwent peritoneal dialysis catheter insertion. In this non-randomized study, 3 groups were formed, and The PD catheter was placed in patients who underwent laparoscopic surgery, laparoscopic omentectomy, and open surgery. Interestingly, the open technique was more effective and successful than the standard laparoscopic method. Contrary to these results, the standard laparoscopic approach is expected to be more effective and successful than the open surgery. Unfortunately, a failure similar to that experienced in standard laparoscopy was the impetus for modification of the approach.

In a meta-analysis of various comparative studies by Gong et al, low-site peritoneal catheter placement was not associated with increased risk of bleeding or infection. Moreover, in the same study, it was reported that short-segment peritoneal catheter placement in the pelvis did not cause bowel movements, and omental wrapping complications were observed less frequently.^[7]^ Our results are consistent with these findings.

International Society for Peritoneal Dialysis guidelines^[6]^ recommend clinical goals^[6]^ for PD access procedures. and are listed in Table 2. The results of modified laparoscopic PD catheter placement can reach the targets specified in the International Society for Peritoneal Dialysis guidelines.

**Table 2:** Comparison of outcomes from modified laparoscopic technique with objectives of ISPD guidelines.

Objectives of ISPD guidelines	Outcomes of modified laparoscopic technique
Catheter patency at 12 months of >95% for advanced placement and >80% for all other catheter insertion methods	92.14%
Exit site/tunnel infection within 30 days of catheter insertion <5%	Nil
Peritonitis within 30 days of catheter insertion <5%	0.38%
Visceral injury (bowel, bladder, solid organ) <1%	Nil
Significant hemorrhage requiring transfusion or surgical intervention <1%	Nil

ISPD = International Society for Peritoneal Dialysis.

### 4.1. Study limitations

This study has some limitations. First, the study consisted of a small sample size, which means that our findings provide preliminary support for the technique but may not be universally applicable or statistically significant due to the limited sample size. Second, this study lacked a comparison group, and there is a possibility that the article may not have included long-term follow-up data, which could account for the potential delayed complications or efficacy of the modified approach.

## 5. Conclusion

This study indicates that the modified laparoscopic method is both safe and effective, with a minimal incidence of complications and a high rate of catheter longevity. However, comparative multicenter studies are needed to obtain strong evidence of the reliability and efficacy of the modified laparoscopic approach.

## Author contributions

**Conceptualization:** Necmi Bayraktar.

**Data curation:** Necmi Bayraktar.

**Formal analysis:** Necmi Bayraktar.

**Investigation:** Necmi Bayraktar.

**Methodology:** Necmi Bayraktar.

**Project administration:** Necmi Bayraktar.

**Resources:** Necmi Bayraktar.

**Supervision:** Fazil Tuncay Aki.

**Validation:** Necmi Bayraktar, Fazil Tuncay Aki.

**Visualization:** Necmi Bayraktar.

**Writing – original draft:** Necmi Bayraktar.

**Writing – review & editing:** Fazil Tuncay Aki.
